# (Pyridine-2,6-diyldimethyl­ene)bis­(diphenyl­methanol)

**DOI:** 10.1107/S1600536808043572

**Published:** 2009-01-08

**Authors:** Wei-Jin Gu, Bing-Xiang Wang

**Affiliations:** a Department of Applied Chemistry, Nanjing Normal University, Nanjing 210097, People’s Republic of China

## Abstract

In the title compound, C_33_H_29_NO_2_, the central pyridyl ring makes dihedral angles of 42.71 (16), 44.78 (16), 85.47 (12) and 76.74 (12)° with the four phenyl rings. There are two intra­molecular O—H⋯N hydrogen bonds. In the crystal structure, mol­ecules are linked into a chain running along the *b* axis by a weak C—H⋯π inter­action.

## Related literature

For organometallic pincer complexes, see: Dupont *et al.* (2005 ▶); Gauvin *et al.* (2001 ▶); Haenel *et al.* (2001 ▶); van der Boom & Milstein (2003 ▶); van der Boom *et al.* (1997 ▶); Vigalok & Milstein (2001 ▶); Bergbreiter *et al.* (1999 ▶). The title compound was prepared according to the procedure described by Berg & Holm (1985 ▶).
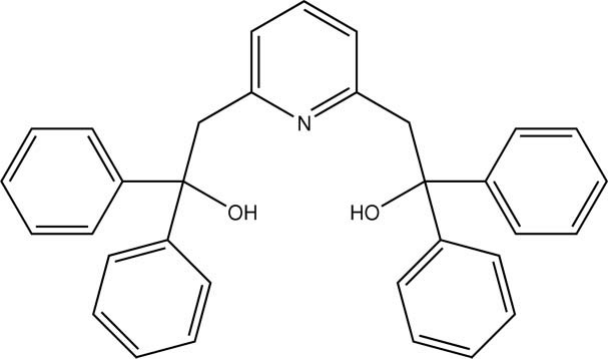


## Experimental

### 

#### Crystal data

C_33_H_29_NO_2_
*M* *_r_* = 471.57
Monoclinic, 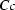

*a* = 18.492 (3) Å
*b* = 10.1039 (17) Å
*c* = 16.097 (3) Å
β = 121.234 (2)°
*V* = 2571.7 (8) Å^3^
*Z* = 4
Mo *K*α radiation
μ = 0.08 mm^−1^
*T* = 291 (2) K
0.30 × 0.26 × 0.24 mm

#### Data collection

Bruker SMART APEX CCD diffractometer
Absorption correction: multi-scan (**SADABS**; Bruker, 2000 ▶) *T*_min_ = 0.980, *T*_max_ = 0.982
10905 measured reflections
2960 independent reflections
2695 reflections with *I* > 2σ(*I*)
*R*_int_ = 0.040

#### Refinement

*R*[*F* ^2^ > 2σ(*F* ^2^)] = 0.054
*wR*(*F* ^2^) = 0.127
*S* = 1.04
2960 reflections
331 parameters
2 restraints
H atoms treated by a mixture of independent and constrained refinement
Δρ_max_ = 0.41 e Å^−3^
Δρ_min_ = −0.39 e Å^−3^

### 

Data collection: *SMART* (Bruker, 2000 ▶); cell refinement: *SAINT* (Bruker, 2000 ▶); data reduction: *SAINT*; program(s) used to solve structure: *SHELXS97* (Sheldrick, 2008 ▶); program(s) used to refine structure: *SHELXL97* (Sheldrick, 2008 ▶); molecular graphics: *SHELXTL* (Sheldrick, 2008 ▶); software used to prepare material for publication: *SHELXTL*.

## Supplementary Material

Crystal structure: contains datablocks global, I. DOI: 10.1107/S1600536808043572/is2375sup1.cif

Structure factors: contains datablocks I. DOI: 10.1107/S1600536808043572/is2375Isup2.hkl

Additional supplementary materials:  crystallographic information; 3D view; checkCIF report

## Figures and Tables

**Table 1:** Hydrogen-bond geometry (Å, °)

*D*—H⋯*A*	*D*—H	H⋯*A*	*D*⋯*A*	*D*—H⋯*A*
O1—H1⋯N1	0.82 (5)	2.34 (5)	3.013 (4)	139 (4)
O2—H2*A*⋯N1	0.82 (5)	2.20 (5)	2.854 (4)	136 (4)
C31—H31⋯*Cg*1^i^	0.93	3.08	3.973 (3)	162

Symmetry code: (i) 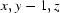. *Cg*1 is the centroid of the C8–C13 ring.

**Fig. 1.**
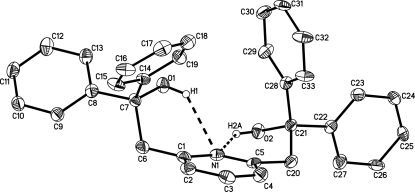
A view of the title compound showing the atom-numbering scheme and displacement ellipsoids drawn at 30% probability level. Dashed lines indicate hydrogen bonds and all H atoms except those involved in hydrogen bonding have been omitted for clarify.

**Fig. 2.**
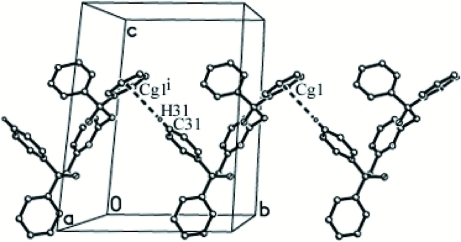
The 1-D chain, viewed along the a axis. Dashed lines indicate the C—H···π interaction between the neighbouring molecules [symmetry code: (i) x, -1 + y, z]. H atoms not involved in the interaction have been omitted for clarify.

## supplementary crystallographic information

### Comment

Currently, organometallic pincer complexes attract much attention because of their widespread applications in catalysis and material sciences (Dupont *et al.*, 2005; van der Boom & Milstein, 2003). Major recent findings have been the generation of efficient dehydrogenation (Haenel *et al.*, 2001) and Heck type catalysts (Bergbreiter *et al.*, 1999), activation of strong C—O (van der Boom *et al.*, 1997) and C—C bonds (Gauvin *et al.*, 2001), and trapping of various intermediates and unusual molecules (Vigalok & Milstein, 2001). 2,6-Bis(2-hydroxy-2,2-diphenylethyl)pyridine, (I), could coordinate with transition metals to form pincer complexes. In our studies, we have got its single crystals and herein reported its crystal structure.

The crystal structure of title compound, C_33_H_29_NO_2_, reveals that all the bond lengths and angles have normal values. Each asymmetric unit in (I) contains four phenyl rings A (C8—C13), B (C14—C19), C (C22—C27) D (C28—C33) and a pyridyl ring E (N1/C1—C5). The rings A, B, C, D and E are all not coplanar, their dihedral angles between rings A and B, B and E, E and C, C and D being 68.13 (15), 44.79 (16), 85.48 (11) and 86.85 (14)°, respectively. The dihedral angles between rings A and E, B and E, C and E, D and E are 42.71 (16), 44.78 (16), 85.47 (12) and 76.74 (12)°, respectively. In the molecule there are two intramolecular O—H···N hydrogen bonds (Table 1 and Fig. 1). In the crystal, there is a weak C—H···π interaction (C31—H31···*Cg*1^i^, i: *x*, -1 + *y*, *z*; *Cg*1 is the centroid of ring A) between the neighbouring molecules (Table 1). Through the weak C—H···π interactions, the one-dimensional chains are formed along the *b* axis (Fig. 2).

### Experimental

2,6-Bis(2-hydroxy-2,2-diphenylethyl)pyridine was prepared by 2,6-lutidine and benzophenone (yield 30%) according to a procedure described in the literature (Berg & Holm, 1985). Colorless crystals were obtained by recrystallized from light petroleum-ethyl acetate (*v*/*v* 5/1) at room temperature.

^1^H-NMR (CDCl_3_, 400 MHz) δ: 7.17–7.37 (21 H, m, 4Ph + 4-H), 6.69 (2 H, d, J = 7.5 Hz, 3-H + 5-H), 5.25 (2 H, s, 2OH), 3.68 (4 H, s, 2CH_2_).

### Refinement

H atoms bonded to O atoms were located in a difference map and their positional parameters were refined with *U*_iso_(H) = 1.2*U*_eq_(O). Other H atoms were positioned geometrically (C—H = 0.93–0.97 Å) and refined using a riding model, with *U*_iso_(H) = 1.2*U*_eq_(C). In the absence of significant anomalous scattering effects, Friedel pairs have been merged.

### Crystal data

**Table:**

C_33_H_29_NO_2_	*F*(000) = 1000
*M**_r_* = 471.57	*D*_x_ = 1.218 Mg m^−^^3^
Monoclinic, *C**c*	Mo *K*α radiation, λ = 0.71073 Å
Hall symbol: C -2yc	Cell parameters from 6093 reflections
*a* = 18.492 (3) Å	θ = 2.4–27.5°
*b* = 10.1039 (17) Å	µ = 0.08 mm^−^^1^
*c* = 16.097 (3) Å	*T* = 291 K
β = 121.234 (2)°	Block, colourless
*V* = 2571.7 (8) Å^3^	0.30 × 0.26 × 0.24 mm
*Z* = 4	

### Data collection

**Table:**

Bruker SMART APEX CCD diffractometer	2960 independent reflections
Radiation source: sealed tube	2695 reflections with *I* > 2σ(*I*)
graphite	*R*_int_ = 0.040
φ and ω scans	θ_max_ = 27.6°, θ_min_ = 2.4°
Absorption correction: multi-scan (*SADABS*; Bruker, 2000)	*h* = −24→21
*T*_min_ = 0.980, *T*_max_ = 0.982	*k* = −13→13
10905 measured reflections	*l* = −20→20

### Refinement

**Table:**

Refinement on *F*^2^	Primary atom site location: structure-invariant direct methods
Least-squares matrix: full	Secondary atom site location: difference Fourier map
*R*[*F*^2^ > 2σ(*F*^2^)] = 0.054	Hydrogen site location: inferred from neighbouring sites
*wR*(*F*^2^) = 0.127	H atoms treated by a mixture of independent and constrained refinement
*S* = 1.04	*w* = 1/[σ^2^(*F*_o_^2^) + (0.05*P*)^2^ + 1.99*P*] where *P* = (*F*_o_^2^ + 2*F*_c_^2^)/3
2960 reflections	(Δ/σ)_max_ < 0.001
331 parameters	Δρ_max_ = 0.41 e Å^−^^3^
2 restraints	Δρ_min_ = −0.39 e Å^−^^3^

### Special details

**Table:**

Geometry. All e.s.d.'s (except the e.s.d. in the dihedral angle between two l.s. planes) are estimated using the full covariance matrix. The cell e.s.d.'s are taken into account individually in the estimation of e.s.d.'s in distances, angles and torsion angles; correlations between e.s.d.'s in cell parameters are only used when they are defined by crystal symmetry. An approximate (isotropic) treatment of cell e.s.d.'s is used for estimating e.s.d.'s involving l.s. planes.Least-squares planes (*x*,*y*,*z* in crystal coordinates) and deviations from them (* indicates atom used to define plane)-2.3376 (0.0303) *x* - 5.1684 (0.0138) *y* + 12.7530 (0.0167) *z* = 0.3615 (0.0248)* 0.0017 (0.0025) C8 * -0.0041 (0.0026) C9 * 0.0000 (0.0028) C10 * 0.0065 (0.0029) C11 * -0.0089 (0.0030) C12 * 0.0048 (0.0028) C13Rms deviation of fitted atoms = 0.005215.5569 (0.0164) *x* - 0.4094 (0.0178) *y* + 0.3974 (0.0262) *z* = 7.3929 (0.0188)Angle to previous plane (with approximate e.s.d.) = 68.13 (0.15)* -0.0126 (0.0027) C14 * 0.0109 (0.0029) C15 * -0.0002 (0.0031) C16 * -0.0092 (0.0030) C17 * 0.0076 (0.0030) C18 * 0.0035 (0.0029) C19Rms deviation of fitted atoms = 0.0085- 8.9504 (0.0218) *x* + 7.2017 (0.0078) *y* - 2.9469 (0.0230) *z* = 2.8842 (0.0118)Angle to previous plane (with approximate e.s.d.) = 44.79 (0.16)* 0.0048 (0.0024) N1 * 0.0001 (0.0031) C1 * 0.0004 (0.0030) C2 * -0.0045 (0.0028) C3 * 0.0087 (0.0031) C4 * -0.0091 (0.0024) C5 * -0.0002 (0.0022) C6Rms deviation of fitted atoms = 0.005416.3880 (0.0176) *x* + 4.6610 (0.0180) *y* - 7.9763 (0.0254) *z* = 6.8874 (0.0075)Angle to previous plane (with approximate e.s.d.) = 85.48 (0.11)* -0.0149 (0.0028) C22 * 0.0006 (0.0031) C23 * 0.0132 (0.0033) C24 * -0.0125 (0.0034) C25 * -0.0023 (0.0036) C26 * 0.0160 (0.0032) C27Rms deviation of fitted atoms = 0.0117- 6.3202 (0.0322) *x* + 6.0939 (0.0141) *y* + 12.7724 (0.0177) *z* = 5.2974 (0.0138)Angle to previous plane (with approximate e.s.d.) = 86.85 (0.14)* 0.0000 (0.0028) C28 * 0.0000 (0.0032) C29 * 0.0000 (0.0033) C30 * 0.0000 (0.0031) C31 * 0.0000 (0.0031) C32 * 0.0000 (0.0029) C33Rms deviation of fitted atoms = 0.0000###############################Least-squares planes (*x*,*y*,*z* in crystal coordinates) and deviations from them (* indicates atom used to define plane)- 8.9516 (0.0242) *x* + 7.2008 (0.0111) *y* - 2.9471 (0.0232) *z* = 2.8832 (0.0159)* 0.0046 (0.0022) N1 * -0.0001 (0.0025) C1 * 0.0003 (0.0027) C2 * -0.0045 (0.0028) C3 * 0.0088 (0.0028) C4 * -0.0091 (0.0025) C5Rms deviation of fitted atoms = 0.0058- 2.3376 (0.0303) *x* - 5.1684 (0.0138) *y* + 12.7530 (0.0167) *z* = 0.3615 (0.0248)Angle to previous plane (with approximate e.s.d.) = 42.71 (0.16)* 0.0017 (0.0025) C8 * -0.0041 (0.0026) C9 * 0.0000 (0.0028) C10 * 0.0065 (0.0029) C11 * -0.0089 (0.0030) C12 * 0.0048 (0.0028) C13Rms deviation of fitted atoms = 0.0052- 8.9516 (0.0242) *x* + 7.2008 (0.0111) *y* - 2.9471 (0.0232) *z* = 2.8832 (0.0159)Angle to previous plane (with approximate e.s.d.) = 42.71 (0.16)* 0.0046 (0.0022) N1 * -0.0001 (0.0025) C1 * 0.0003 (0.0027) C2 * -0.0045 (0.0028) C3 * 0.0088 (0.0028) C4 * -0.0091 (0.0025) C5Rms deviation of fitted atoms = 0.005815.5569 (0.0164) *x* - 0.4094 (0.0178) *y* + 0.3974 (0.0262) *z* = 7.3929 (0.0188)Angle to previous plane (with approximate e.s.d.) = 44.78 (0.16)* -0.0126 (0.0027) C14 * 0.0109 (0.0029) C15 * -0.0002 (0.0031) C16 * -0.0092 (0.0030) C17 * 0.0076 (0.0030) C18 * 0.0035 (0.0029) C19Rms deviation of fitted atoms = 0.0085- 8.9516 (0.0242) *x* + 7.2008 (0.0111) *y* - 2.9471 (0.0232) *z* = 2.8832 (0.0159)Angle to previous plane (with approximate e.s.d.) = 44.78 (0.16)* 0.0046 (0.0022) N1 * -0.0001 (0.0025) C1 * 0.0003 (0.0027) C2 * -0.0045 (0.0028) C3 * 0.0088 (0.0028) C4 * -0.0091 (0.0025) C5Rms deviation of fitted atoms = 0.005816.3880 (0.0176) *x* + 4.6610 (0.0180) *y* - 7.9763 (0.0254) *z* = 6.8874 (0.0075)Angle to previous plane (with approximate e.s.d.) = 85.47 (0.12)* -0.0149 (0.0028) C22 * 0.0006 (0.0031) C23 * 0.0132 (0.0033) C24 * -0.0125 (0.0034) C25 * -0.0023 (0.0036) C26 * 0.0160 (0.0032) C27Rms deviation of fitted atoms = 0.0117- 8.9516 (0.0242) *x* + 7.2008 (0.0111) *y* - 2.9471 (0.0232) *z* = 2.8832 (0.0159)Angle to previous plane (with approximate e.s.d.) = 85.47 (0.12)* 0.0046 (0.0022) N1 * -0.0001 (0.0025) C1 * 0.0003 (0.0027) C2 * -0.0045 (0.0028) C3 * 0.0088 (0.0028) C4 * -0.0091 (0.0025) C5Rms deviation of fitted atoms = 0.0058- 6.3202 (0.0322) *x* + 6.0939 (0.0141) *y* + 12.7724 (0.0177) *z* = 5.2974 (0.0138)Angle to previous plane (with approximate e.s.d.) = 76.74 (0.12)* 0.0000 (0.0028) C28 * 0.0000 (0.0032) C29 * 0.0000 (0.0033) C30 * 0.0000 (0.0031) C31 * 0.0000 (0.0031) C32 * 0.0000 (0.0029) C33Rms deviation of fitted atoms = 0.0000
Refinement. Refinement of *F*^2^ against ALL reflections. The weighted *R*-factor *wR* and goodness of fit *S* are based on *F*^2^, conventional *R*-factors *R* are based on *F*, with *F* set to zero for negative *F*^2^. The threshold expression of *F*^2^ > σ(*F*^2^) is used only for calculating *R*-factors(gt) *etc*. and is not relevant to the choice of reflections for refinement. *R*-factors based on *F*^2^ are statistically about twice as large as those based on *F*, and *R*- factors based on ALL data will be even larger.

### Fractional atomic coordinates and isotropic or equivalent isotropic displacement parameters (Å^2^)

**Table:**

	*x*	*y*	*z*	*U*_iso_*/*U*_eq_	
C1	0.3187 (2)	0.9740 (4)	0.4335 (3)	0.0489 (8)	
C2	0.2728 (3)	0.9343 (4)	0.4758 (3)	0.0537 (9)	
H2	0.2837	0.9721	0.5339	0.064*	
C3	0.2110 (3)	0.8385 (4)	0.4312 (3)	0.0548 (10)	
H3	0.1804	0.8102	0.4589	0.066*	
C4	0.1957 (3)	0.7858 (5)	0.3442 (3)	0.0563 (10)	
H4	0.1535	0.7228	0.3115	0.068*	
C5	0.2441 (2)	0.8281 (4)	0.3068 (2)	0.0427 (7)	
C6	0.3866 (3)	1.0774 (4)	0.4800 (3)	0.0526 (9)	
H6A	0.3791	1.1265	0.5269	0.063*	
H6B	0.3803	1.1391	0.4305	0.063*	
C7	0.4777 (2)	1.0188 (3)	0.5323 (2)	0.0416 (7)	
C8	0.5447 (2)	1.1246 (4)	0.5841 (2)	0.0443 (8)	
C9	0.5332 (3)	1.2363 (4)	0.6268 (3)	0.0535 (9)	
H9	0.4815	1.2498	0.6223	0.064*	
C10	0.5985 (3)	1.3284 (4)	0.6764 (3)	0.0532 (9)	
H10	0.5901	1.4024	0.7047	0.064*	
C11	0.6736 (3)	1.3097 (4)	0.6831 (3)	0.0591 (11)	
H11	0.7171	1.3705	0.7169	0.071*	
C12	0.6865 (3)	1.2012 (4)	0.6403 (3)	0.0581 (11)	
H12	0.7380	1.1906	0.6439	0.070*	
C13	0.6231 (3)	1.1077 (4)	0.5919 (3)	0.0542 (9)	
H13	0.6327	1.0338	0.5645	0.065*	
C14	0.4830 (2)	0.9113 (4)	0.6037 (3)	0.0428 (7)	
C15	0.4833 (3)	0.9465 (4)	0.6872 (3)	0.0518 (9)	
H15	0.4867	1.0353	0.7039	0.062*	
C16	0.4785 (3)	0.8508 (4)	0.7464 (3)	0.0547 (10)	
H16	0.4777	0.8753	0.8016	0.066*	
C17	0.4751 (3)	0.7177 (4)	0.7218 (3)	0.0584 (11)	
H17	0.4713	0.6525	0.7601	0.070*	
C18	0.4773 (3)	0.6844 (4)	0.6424 (3)	0.0603 (10)	
H18	0.4763	0.5953	0.6275	0.072*	
C19	0.4810 (3)	0.7780 (4)	0.5822 (3)	0.0574 (10)	
H19	0.4823	0.7518	0.5275	0.069*	
C20	0.2248 (2)	0.7717 (4)	0.2092 (3)	0.0482 (8)	
H20A	0.1883	0.8332	0.1582	0.058*	
H20B	0.1938	0.6895	0.1974	0.058*	
C21	0.3036 (2)	0.7445 (3)	0.2015 (2)	0.0392 (7)	
C22	0.2752 (2)	0.6821 (4)	0.1024 (2)	0.0410 (7)	
C23	0.3043 (3)	0.5634 (4)	0.0909 (3)	0.0542 (10)	
H23	0.3442	0.5168	0.1454	0.065*	
C24	0.2749 (3)	0.5108 (5)	−0.0018 (3)	0.0609 (11)	
H24	0.2961	0.4306	−0.0084	0.073*	
C25	0.2159 (3)	0.5761 (4)	−0.0816 (3)	0.0561 (10)	
H25	0.1949	0.5396	−0.1430	0.067*	
C26	0.1877 (3)	0.6941 (5)	−0.0720 (3)	0.0631 (12)	
H26	0.1476	0.7396	−0.1271	0.076*	
C27	0.2175 (3)	0.7490 (4)	0.0192 (3)	0.0602 (11)	
H27	0.1985	0.8320	0.0243	0.072*	
C28	0.3695 (2)	0.6555 (3)	0.2848 (2)	0.0383 (7)	
C29	0.4551 (2)	0.6722 (4)	0.3192 (3)	0.0573 (10)	
H29	0.4729	0.7406	0.2954	0.069*	
C30	0.5141 (3)	0.5866 (4)	0.3892 (3)	0.0618 (11)	
H30	0.5714	0.5978	0.4123	0.074*	
C31	0.4874 (3)	0.4844 (4)	0.4249 (3)	0.0521 (10)	
H31	0.5269	0.4271	0.4717	0.063*	
C32	0.4018 (3)	0.4677 (5)	0.3905 (3)	0.0618 (12)	
H32	0.3840	0.3992	0.4143	0.074*	
C33	0.3429 (2)	0.5532 (4)	0.3204 (3)	0.0470 (9)	
H33	0.2856	0.5421	0.2974	0.056*	
N1	0.3029 (2)	0.9203 (3)	0.3487 (2)	0.0467 (7)	
O1	0.49165 (17)	0.9616 (3)	0.46054 (19)	0.0497 (6)	
H1	0.451 (3)	0.917 (5)	0.423 (4)	0.060*	
O2	0.34180 (18)	0.8688 (3)	0.2017 (2)	0.0503 (6)	
H2A	0.352 (3)	0.911 (5)	0.250 (4)	0.060*	

### Atomic displacement parameters (Å^2^)

**Table:**

	*U*^11^	*U*^22^	*U*^33^	*U*^12^	*U*^13^	*U*^23^
C1	0.054 (2)	0.0442 (19)	0.0435 (19)	0.0163 (16)	0.0219 (17)	0.0076 (15)
C2	0.052 (2)	0.066 (2)	0.048 (2)	0.0145 (19)	0.0301 (19)	−0.0020 (18)
C3	0.060 (2)	0.059 (2)	0.065 (2)	0.0100 (19)	0.046 (2)	0.008 (2)
C4	0.052 (2)	0.063 (2)	0.056 (2)	−0.0030 (18)	0.0294 (19)	0.0000 (19)
C5	0.0350 (16)	0.0516 (19)	0.0351 (16)	0.0133 (14)	0.0136 (14)	0.0095 (14)
C6	0.060 (2)	0.0430 (19)	0.044 (2)	0.0092 (17)	0.0187 (18)	0.0022 (15)
C7	0.0529 (19)	0.0390 (16)	0.0342 (16)	0.0037 (14)	0.0235 (15)	−0.0013 (13)
C8	0.053 (2)	0.0441 (18)	0.0312 (16)	−0.0001 (15)	0.0186 (15)	0.0053 (14)
C9	0.061 (2)	0.049 (2)	0.0378 (19)	0.0057 (17)	0.0163 (17)	−0.0007 (15)
C10	0.066 (2)	0.0429 (19)	0.043 (2)	0.0024 (17)	0.0228 (18)	−0.0019 (15)
C11	0.058 (2)	0.049 (2)	0.053 (2)	−0.0167 (18)	0.017 (2)	−0.0018 (18)
C12	0.070 (3)	0.055 (2)	0.057 (2)	−0.023 (2)	0.038 (2)	−0.0020 (18)
C13	0.056 (2)	0.050 (2)	0.058 (2)	−0.0109 (17)	0.030 (2)	−0.0053 (18)
C14	0.0409 (17)	0.0477 (18)	0.0437 (18)	0.0091 (14)	0.0248 (15)	0.0041 (14)
C15	0.066 (2)	0.054 (2)	0.062 (2)	0.0202 (18)	0.052 (2)	0.0165 (18)
C16	0.056 (2)	0.069 (2)	0.063 (2)	0.0193 (19)	0.047 (2)	0.030 (2)
C17	0.059 (2)	0.061 (2)	0.059 (2)	−0.0047 (19)	0.034 (2)	0.035 (2)
C18	0.054 (2)	0.054 (2)	0.061 (3)	−0.0055 (19)	0.021 (2)	0.011 (2)
C19	0.061 (2)	0.0410 (18)	0.062 (2)	−0.0001 (17)	0.026 (2)	0.0063 (18)
C20	0.0379 (17)	0.053 (2)	0.0414 (19)	−0.0019 (15)	0.0122 (15)	0.0011 (16)
C21	0.0383 (16)	0.0383 (16)	0.0353 (16)	−0.0021 (13)	0.0151 (14)	0.0014 (13)
C22	0.0394 (17)	0.0510 (19)	0.0329 (16)	−0.0028 (14)	0.0189 (14)	0.0014 (14)
C23	0.049 (2)	0.068 (3)	0.0390 (19)	0.0143 (19)	0.0181 (17)	−0.0059 (17)
C24	0.056 (2)	0.068 (3)	0.057 (2)	0.013 (2)	0.028 (2)	−0.026 (2)
C25	0.064 (2)	0.057 (2)	0.048 (2)	0.0025 (18)	0.030 (2)	−0.0255 (17)
C26	0.060 (2)	0.072 (3)	0.043 (2)	0.036 (2)	0.0165 (19)	0.003 (2)
C27	0.062 (2)	0.056 (2)	0.043 (2)	−0.0024 (19)	0.0134 (19)	0.0019 (17)
C28	0.0431 (17)	0.0413 (16)	0.0285 (14)	−0.0007 (13)	0.0172 (13)	0.0062 (12)
C29	0.0421 (19)	0.061 (2)	0.052 (2)	−0.0080 (17)	0.0125 (17)	0.0114 (18)
C30	0.0364 (19)	0.064 (2)	0.067 (3)	−0.0021 (17)	0.0139 (19)	0.005 (2)
C31	0.060 (2)	0.057 (2)	0.0392 (17)	0.0355 (18)	0.0257 (17)	0.0174 (16)
C32	0.069 (3)	0.072 (3)	0.061 (2)	0.037 (2)	0.046 (2)	0.041 (2)
C33	0.0476 (18)	0.060 (2)	0.057 (2)	0.0232 (16)	0.0431 (18)	0.0297 (18)
N1	0.0441 (16)	0.0486 (17)	0.0441 (16)	0.0099 (13)	0.0205 (13)	0.0102 (13)
O1	0.0531 (15)	0.0579 (16)	0.0533 (15)	−0.0102 (12)	0.0382 (13)	−0.0174 (13)
O2	0.0600 (16)	0.0400 (13)	0.0445 (14)	−0.0098 (12)	0.0226 (13)	0.0057 (11)

### Geometric parameters (Å, °)

**Table:**

C1—N1	1.353 (5)	C17—H17	0.9300
C1—C2	1.394 (6)	C18—C19	1.382 (6)
C1—C6	1.501 (6)	C18—H18	0.9300
C2—C3	1.381 (6)	C19—H19	0.9300
C2—H2	0.9300	C20—C21	1.549 (5)
C3—C4	1.384 (6)	C20—H20A	0.9700
C3—H3	0.9300	C20—H20B	0.9700
C4—C5	1.381 (5)	C21—O2	1.440 (4)
C4—H4	0.9300	C21—C22	1.533 (5)
C5—N1	1.321 (5)	C21—C28	1.548 (5)
C5—C20	1.530 (5)	C22—C23	1.366 (5)
C6—C7	1.557 (5)	C22—C27	1.381 (5)
C6—H6A	0.9700	C23—C24	1.401 (5)
C6—H6B	0.9700	C23—H23	0.9300
C7—O1	1.431 (4)	C24—C25	1.351 (6)
C7—C8	1.518 (5)	C24—H24	0.9300
C7—C14	1.547 (5)	C25—C26	1.342 (5)
C8—C9	1.394 (5)	C25—H25	0.9300
C8—C13	1.399 (6)	C26—C27	1.388 (6)
C9—C10	1.401 (6)	C26—H26	0.9300
C9—H9	0.9300	C27—H27	0.9300
C10—C11	1.350 (6)	C28—C29	1.390 (5)
C10—H10	0.9300	C28—C33	1.390 (4)
C11—C12	1.380 (6)	C29—C30	1.390 (6)
C11—H11	0.9300	C29—H29	0.9300
C12—C13	1.388 (5)	C30—C31	1.390 (6)
C12—H12	0.9300	C30—H30	0.9300
C13—H13	0.9300	C31—C32	1.390 (6)
C14—C19	1.387 (5)	C31—H31	0.9300
C14—C15	1.387 (5)	C32—C33	1.390 (5)
C15—C16	1.392 (5)	C32—H32	0.9300
C15—H15	0.9300	C33—H33	0.9300
C16—C17	1.394 (6)	O1—H1	0.82 (5)
C16—H16	0.9300	O2—H2A	0.82 (5)
C17—C18	1.342 (7)		
			
N1—C1—C2	120.6 (4)	C17—C18—C19	122.3 (4)
N1—C1—C6	118.0 (4)	C17—C18—H18	118.9
C2—C1—C6	121.4 (4)	C19—C18—H18	118.9
C3—C2—C1	119.8 (4)	C18—C19—C14	119.5 (4)
C3—C2—H2	120.1	C18—C19—H19	120.2
C1—C2—H2	120.1	C14—C19—H19	120.2
C2—C3—C4	118.4 (4)	C5—C20—C21	114.8 (3)
C2—C3—H3	120.8	C5—C20—H20A	108.6
C4—C3—H3	120.8	C21—C20—H20A	108.6
C5—C4—C3	118.9 (4)	C5—C20—H20B	108.6
C5—C4—H4	120.5	C21—C20—H20B	108.6
C3—C4—H4	120.5	H20A—C20—H20B	107.5
N1—C5—C4	122.9 (4)	O2—C21—C22	105.3 (3)
N1—C5—C20	118.7 (3)	O2—C21—C28	109.9 (3)
C4—C5—C20	118.3 (4)	C22—C21—C28	110.7 (3)
C1—C6—C7	113.3 (3)	O2—C21—C20	109.0 (3)
C1—C6—H6A	108.9	C22—C21—C20	109.0 (3)
C7—C6—H6A	108.9	C28—C21—C20	112.6 (3)
C1—C6—H6B	108.9	C23—C22—C27	117.3 (4)
C7—C6—H6B	108.9	C23—C22—C21	123.8 (3)
H6A—C6—H6B	107.7	C27—C22—C21	119.0 (3)
O1—C7—C8	106.9 (3)	C22—C23—C24	121.0 (4)
O1—C7—C14	110.4 (3)	C22—C23—H23	119.5
C8—C7—C14	111.6 (3)	C24—C23—H23	119.5
O1—C7—C6	108.3 (3)	C25—C24—C23	120.3 (4)
C8—C7—C6	112.1 (3)	C25—C24—H24	119.9
C14—C7—C6	107.6 (3)	C23—C24—H24	119.9
C9—C8—C13	118.1 (4)	C26—C25—C24	119.7 (4)
C9—C8—C7	123.4 (3)	C26—C25—H25	120.2
C13—C8—C7	118.4 (3)	C24—C25—H25	120.2
C8—C9—C10	120.8 (4)	C25—C26—C27	120.8 (4)
C8—C9—H9	119.6	C25—C26—H26	119.6
C10—C9—H9	119.6	C27—C26—H26	119.6
C11—C10—C9	120.0 (4)	C22—C27—C26	120.9 (4)
C11—C10—H10	120.0	C22—C27—H27	119.5
C9—C10—H10	120.0	C26—C27—H27	119.5
C10—C11—C12	120.5 (4)	C29—C28—C33	120.0 (3)
C10—C11—H11	119.7	C29—C28—C21	119.8 (3)
C12—C11—H11	119.7	C33—C28—C21	120.1 (3)
C11—C12—C13	120.5 (4)	C28—C29—C30	120.0 (4)
C11—C12—H12	119.7	C28—C29—H29	120.0
C13—C12—H12	119.7	C30—C29—H29	120.0
C12—C13—C8	120.0 (4)	C31—C30—C29	120.0 (4)
C12—C13—H13	120.0	C31—C30—H30	120.0
C8—C13—H13	120.0	C29—C30—H30	120.0
C19—C14—C15	118.6 (4)	C30—C31—C32	120.0 (3)
C19—C14—C7	120.8 (3)	C30—C31—H31	120.0
C15—C14—C7	120.5 (3)	C32—C31—H31	120.0
C14—C15—C16	121.0 (4)	C33—C32—C31	120.0 (4)
C14—C15—H15	119.5	C33—C32—H32	120.0
C16—C15—H15	119.5	C31—C32—H32	120.0
C15—C16—C17	119.0 (4)	C32—C33—C28	120.0 (3)
C15—C16—H16	120.5	C32—C33—H33	120.0
C17—C16—H16	120.5	C28—C33—H33	120.0
C18—C17—C16	119.6 (3)	C5—N1—C1	119.2 (3)
C18—C17—H17	120.2	C7—O1—H1	109 (3)
C16—C17—H17	120.2	C21—O2—H2A	109 (3)

### Hydrogen-bond geometry (Å, °)

**Table:**

*D*—H···*A*	*D*—H	H···*A*	*D*···*A*	*D*—H···*A*
O1—H1···N1	0.82 (5)	2.34 (5)	3.013 (4)	139 (4)
O2—H2A···N1	0.82 (5)	2.20 (5)	2.854 (4)	136 (4)
C31—H31···Cg1^i^	0.93	3.08	3.973 (3)	162

Symmetry codes: (i) *x*, *y*−1, *z*.
